# Magnification inferred curvature for real-time curvature monitoring

**DOI:** 10.1038/s41598-021-88722-6

**Published:** 2021-04-30

**Authors:** Alexandre Arnoult, Jonathan Colin

**Affiliations:** LAAS-CNRS, Université de Toulouse, CNRS, Toulouse, France

**Keywords:** Optical techniques, Characterization and analytical techniques

## Abstract

The in situ and real-time measurement of curvature changes of optically reflecting surfaces is a key element to better control bottom-up fabrication processes in the semiconductor industry, but also to follow or adjust mirror deformations during fabrication and use for space or optics industries. Despite progresses made in the last two decades thanks to laser deflectometry-based techniques, the community lacks an instrument, easy to use, robust to tough environments and easily compatible with a large range of fabrication processes. We describe here a new method, called magnification inferred curvature (MIC), based on the determination of the magnification factor of the virtual image size of a known object created by a reflecting curved surface (the substrate) acting as a spherical mirror. The optical formalism, design, and proof of concept are presented. The precision, accuracy, and advantages of the MIC method are illustrated from selected examples taken from real-time growth monitoring and compared with state-of-the-art laser deflectometry-based instruments.

## Introduction

Precise and accurate determination of the curvature^1^ of an optically reflecting surface remains a challenge to build key technological components in a large range of industrial sectors, such as giant mirrors for space industry, small mirrors for lasers applications, determination of eye curvature in ophthalmology or wafers in the semiconductor industry. Many techniques such as interferometry (Zygo), stylus or optical surface profilometry (KLA-Tencor), confocal microscopy (Sensofar), Shack-Hartmann Wave Front Sensing (Imagine Optics, Phasics, Lambda-X) or laser deflectometry (kSA, Laytec) are commercially available to measure wafer curvature^2–6^. However, only laser deflectometry-based techniques give access to real-time and in situ measurement of theses curvatures during fabrication processes, by either measuring the deflection of a single laser beam^7^ or the relative separation between several reflected beams^8^. Chason and Floro^9,10^ have extended this method, mainly by measuring the relative spacing of reflected laser spots by a sensor matrix, usually a CCD. During the last two decades, their Multi-Beam Optical Stress Sensor (MOSS), has been a key element to unravel many atomistic phenomena driving nucleation and growth processes and understanding the development of stress during growth of epitaxial and polycrystalline thin films^10–16^.

In the semiconductor industry, challenges such as decreasing the size of building blocks, gaining control on fabrication processes to cut down waste in production and achieving automation of the fabrication process (towards the 4.0 industrial revolution) lack characterization tools that would be easy-to-use, very accurate, and compatible with industrial constraints. Currently, the development of very complex semiconductor heterostructures for optoelectronic components such as Vertical-Cavity Surface-Emitting Lasers (VCSELs)^17,18^, stress-free thick quaternary alloys for high efficiency solar cells^19,20^, mirrors for giant interferometers^21^, microcavities for Bose–Einstein Condensation (BEC)^22,23^ and polariton lasers^24,25^, needs a very accurate in situ determination of alloys composition, layers thicknesses and stress build-up. These new nanotechnologies require industrial growth conditions that make it more challenging to monitor than in academic research. In most applications, the wafer condensing the molecular flux has to rotate (typically 10 – 30 rpm) to ensure film homogeneity, challenging alignment sensitive tools like laser deflectometry-based systems. The use of very thin wafers for specific studies, such as 100 µm thick Si wafers, increases the variation of their curvature as a function of stress, as shown by Stoney’s equation (Eq. (1)). However, the standard thicknesses used in the semiconductor industry are larger, varying from 279 ± 25 µm for 50 mm Si wafers to 775 ± 20 µm for 300 mm Si wafers (SEMI Standard), highlighting the need for an even more sensitive curvature measurement tool. Moreover, when growing a layer of a material which refractive index is different from the underlayer’s, oscillations in the reflectivity of the film occur^26^, which may fall down, for a given wavelength, corresponding to destructive interferences^26,27^. If one uses a monochromatic source to measure the reflectivity, as involved for laser deflectometry-based techniques, the detector might lose the reflected light when reflectivity is low, and the system will be blind at this stage. A white light source would be an easy way to overcome this issue, but is not compatible with the physics of the deflectometry-based technique. With those constraints, laser deflectometry-based systems are limited to monitor accurately the curvature in real-time.

Here we report on a new technique, called MIC for Magnification Inferred Curvature, in which the curvature of a substrate (acting as a curved mirror) is derived from the magnification factor of the virtual image of an object seen through it. This new technique, can overcome most of the limitations of laser deflectometry-based systems, and pushes further the frontier in curvature measurement precision and accuracy. As this new technique is based on an entirely original optical formalism, we provide here the corresponding formalism to calculate the curvature of a spherical mirror at any incidence angle, i.e. outside Gauss’ conditions, which have been carefully validated using Zemax OpticStudio ray-tracing simulations. Design and algorithm’s key elements of the MIC tool are presented and some selected experiments performed using the MIC technique are given to demonstrate and discuss its capabilities in regard of the most precise laser deflectometry-based equipment.

## Method

### Optical formalism

When a film is deposited on an unclamped wafer, stress build-up bends it. Measuring the wafer curvature during thin film deposition allows for a quantitative measurement of the film’s stress. The phenomenological Stoney’s equation (Eq. (1)) links the mean stress $\sigma_{f}$ in the film to the radius of curvature $\overline{R}$ or the curvature $\overline{\kappa } = 1/\overline{R}$, through the following expression:

1 $$\sigma_{f} \cong \frac{{M_{s} h_{s}^{2} }}{{6 \overline{R} h_{f} }} = \overline{\kappa }\frac{{M_{s} h_{s}^{2} }}{{6 h_{f} }}$$

where *h*_*s*_ and *M*_*s*_ are the substrate thickness and biaxial modulus respectively, and *h*_*f*_ is the film thickness. Stoney’s equation also reports that a uniform film giving a uniform stress on/in another material will lead, at a first extent, to a spherical deformation^28^.

**Figure 1:**
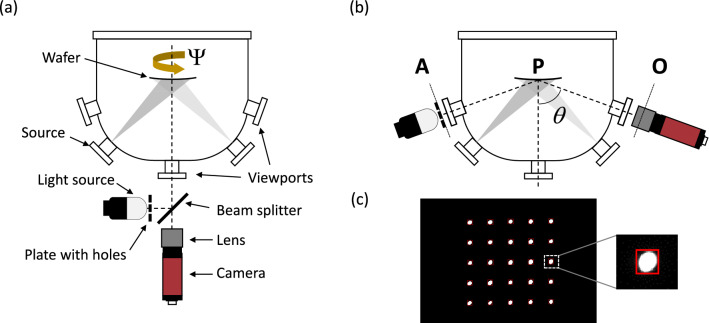
Experimental configuration of the MIC system mounted on a MBE chamber in order to measure the curvature of a wafer in situ and in real-time during MBE growth (**a**,**b**). The system can be mounted either in normal incidence (**a**) or in tilted specular reflection incidence (**b**). The position of the object plane (A), the pole of the reflecting surface (P), and the focal plane of the objective lens (O) are noted in (**b**). (**c**) Shows a typical virtual image of a 1.6 × 1.6 cm^2^ object captured with the camera, where our software tracks the positions of luminous spots forming a 5 × 5 matrix (red rectangles).

An optically reflecting surface acts as a mirror, thus deforming the image of an object it creates. Let A and B be two points of an object. The ratio of the image size $\overline{A^{\prime}B^{\prime}}$ on the physical size of the object itself $\overline{AB}$ is the magnification factor $\gamma$. For a flat mirror, $\gamma = 1$. For a spherical mirror, in Gauss’ conditions, the conjugate equation^29^ leads directly to:

2 $$\gamma = \frac{{\overline{{A^{\prime}B^{\prime}}} }}{{\overline{AB} }} = \frac{1}{{1 - 2\frac{{\overline{AP} }}{{\overline{R}}}}}$$

where P is the pole of the spherical mirror (Fig. 1b).

The curvature $\overline{{\kappa_{ \bot } }} = 1/\overline{R}$ is then, at normal incidence (*θ* = 0°) (Fig. 1a):

3 $$\overline{{\kappa_{ \bot } }} = \frac{1}{{2\overline{AP} }}\frac{\gamma - 1}{\gamma }$$

A way to measure $\overline{{\kappa_{ \bot } }}$ is to capture the image of the object created by the spherically curved surface of interest (substrate) with an optical device (namely a camera and its objective), and compare its size *d* with the size *d*_*0*_ of the image of the same object obtained when the spherical surface curvature is zero (i.e. a flat substrate). Let $\gamma_{c}$ be this pseudo-magnification factor.

4 $$\gamma_{c} = \frac{d}{{d_{0} }}$$

Using geometrical optics considerations, it is straightforward to find that the curvature of the spherical surface $\overline{{\kappa_{ \bot } }}$ in Gauss’ conditions, at normal incidence is:

5 $$\overline{{\kappa_{ \bot } }} = \frac{1}{{2\overline{AP} }}\frac{{\gamma_{C} - 1}}{{\gamma_{C} }} \times \frac{{\overline{AP} + \overline{OP} }}{{\overline{OP} }}$$

where $\overline{OP}$ is the distance between the objective lens focal plane to the mirror’s Pole (Fig. 1b).

When the object and the camera are mounted in a symmetrical geometry with respect to the normal of the substrate surface (Fig. 1b), the effective radius of curvature $R_{e}$ for a spherical mirror is $R_{e - t} = R \times \cos \theta$ in the tangential plane (i.e. directions perpendicular to incidence plane), and $R_{e - s} = R/\cos \theta$ in the sagittal plane^30,31^ (i.e. directions parallel to the incidence plane). At an incidence angle $\theta$, the curvature $\overline{\kappa }\left( \theta \right)$ is inferred from the measurement of the magnification $\gamma_{Ct}$ or $\gamma_{Cs}$ respectively in tangential (t) and sagittal (s) planes using the following equations:

6 $$\overline{{\kappa_{t} }} \left( \theta \right) = \frac{1}{{2\overline{AP} }}\frac{{\gamma_{Ct} - 1}}{{\gamma_{Ct} }}\frac{{\overline{OP} + \overline{AP} }}{{\overline{OP} }}\frac{1}{\cos \theta }$$

7 $$\overline{{\kappa_{s} }} \left( \theta \right) = \frac{1}{{2\overline{AP} }}\frac{{\gamma_{Cs} - 1}}{{\gamma_{Cs} }}\frac{{\overline{OP} + \overline{AP} }}{{\overline{OP} }}\cos \theta$$

If one ignores the precise value of $\theta$, or if this angle varies during the measurement process for any reason, it is possible to extract the geometric mean value of $\overline{\kappa }$ by combining the measurements in sagittal and tangential directions. As long as the deformation of the measured surface is isotropic, this geometric mean curvature at any incidence angle $\theta$ is given by:

8 $$\overline{\left| \kappa \right|} = \sqrt {\overline{{\kappa_{t} }} \left( \theta \right)\overline{{\kappa_{s} }} \left( \theta \right)} = \frac{1}{{2\left| {\overline{AP} } \right|}}\frac{{\left| {\overline{OP} + \overline{AP} } \right|}}{{\left| {\overline{OP} } \right|}}\sqrt {\frac{{\left( {\gamma_{Ct} - 1} \right)\left( {\gamma_{Cs} - 1} \right)}}{{\gamma_{Ct} \gamma_{Cs} }}}$$

Combining Eqs. (1) and (5), for a homogeneous spherical deformation, the product of the stress by the thickness is derived in Eq. (9):

9 $$\sigma_{f} h_{f} \cong M_{s} h_{s}^{2} \frac{{\overline{AP} + \overline{OP} }}{{12\overline{AP} \times \overline{OP} }}\frac{{\gamma_{C} - 1}}{{\gamma_{C} }}$$

### Numerical validation of the model

**Figure 2:**
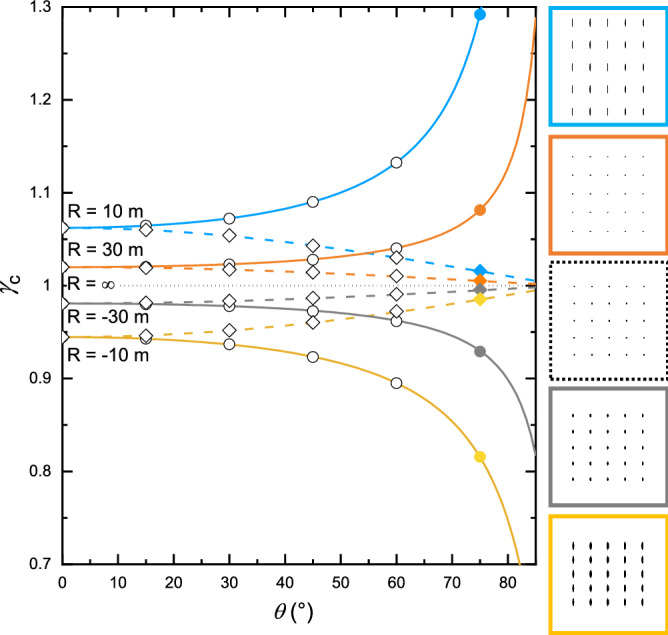
Captured magnification *γ*_*c*_ calculated (curves) and extracted (points) by our image analysis algorithm out of images simulated with Zemax OpticStudio as a function of tilt angle *θ* and for radii of curvature of 10 m (blue), 30 m (orange), infinite (dotted dark), − 30 m (grey) and − 10 m (yellow). The distances $\overline{OP}$ and $\overline{AP}$ used for calculation and simulation are those of our MBE 412 system’s configuration (0.516 m and 0.678 m respectively). The curves show calculation in sagittal (solid) and tangential (dash) directions thanks to Eqs. (6) and (7). At the right hand side are shown inverted color images simulated by Zemax OpticStudio from which plain colored points data are extracted in the figure (for *θ* = 75°). For these images, the sagittal direction is vertical and the tangential direction is horizontal. From top to bottom, these images correspond to 10 m, 30 m, infinite, − 30 m and − 10 m radii of curvature.

In order to check the validity of Eqs. (6) and (7) at any given angle *θ* outside Gauss’ conditions, we have created a numerical model of the MIC setup using the Zemax OpticStudio ray tracing software in non-sequential mode. The substrate is simulated by a mirror surface with a given radius of curvature. The model simulates images captured by a virtual camera composed of a lens, a diaphragm, and a rectangle detector with the same pixel size and density than a standard camera detector, for different reflecting surface radii of curvature and incidence angles *θ* (Fig. 1b). The object is made out of a 4 mm pitch, 5 × 5 matrix of luminous white spots. No mathematical model is introduced in the simulation, as only reflection and refraction laws occur for each simulated ray. One million rays are generated in a 0.1° solid angle towards the chief ray direction for each luminous spot of the 5 × 5 matrix. The numerical images (Fig. 2—right) are then analyzed with our algorithm measuring the centroid (i.e. the chief ray) positions of the luminous spots in the simulated image, and thus their mean relative separation in the tangential and sagittal directions. These separations are compared to those measured in the reference image obtained with a zero curvature (flat) reflecting surface, in order to calculate pseudo-magnification factors γ_Ct_ and γ_Cs_. Curvatures inferred from this image analysis (dots in Fig. 2) are derived from Eqs. (6) and (7) in which the geometrical parameters *θ*, $\overline{AP}$ and $\overline{OP}$ are set to those of the Zemax OpticStudio numerical model. A perfect correlation with a relative error of about 2 × 10^–3^% at most is observed, at any incidence angle, between the curvatures deduced from the simulated images thanks to Eqs. (6) and (7) and the curvatures of the mirror surface introduced in the ray tracing simulation. This validates the equations from the optical formalism reported here and the methodology, precision and accuracy of our image analysis algorithm.

### The curvature measurement tool

The patent pending^32^ experimental setup presented here has been standardized and is now commercially available as the EZ-CURVE system (RIBER S.A), dedicated to follow in situ and in real-time curvature changes during thin film processes under vacuum environments. We have thoroughly developed and tested it on a Riber MBE412 molecular beam epitaxy (MBE) chamber, but it can be readily implemented to other environments like sputter-deposition chambers, or CVD reactors (tested but not reported here). The EZ-CURVE system is made of a luminous object, a camera with an objective and an analyzing software. The luminous object is a matrix of luminous spots, made out of a white light source and an opaque disk in which an array of holes has been drilled. The system can be mounted to either a single viewport at normal incidence (Fig. 1a), or two symmetric viewports (Fig. 1b). In the single viewport configuration, the viewport is facing the wafer at normal incidence, and a beam splitter is used so the camera sees only the virtual image of the luminous object. We developed a dedicated software to precisely measure the centroid positions of the spots from the images acquired by the camera, even in potentially luminous or vibrating environments like plasma deposition or plasma etching chambers. Their mean relative distances in two orthogonal directions are extracted, and compared to reference distances measured from the image created by a flat or a reference surface, with a very fast acquisition and processing rate (100 Hz). Measuring relative positions increases the robustness to mechanical vibrations^10^, and by extent the precision of the MIC technique.

The system can be calibrated either on a commercially available reference mirror with a known curvature for absolute measurement or on the starting surface (which is then considered as the reference) for relative measurements. The latter is only possible because the relationship between *γ*_*c*_ and $\overline{\kappa }$ is linear for relatively small curvatures:

10 $$\gamma_{c} = \frac{1}{{1 - 2\overline{\kappa } \frac{{\overline{AP} \times \overline{OP} }}{{\overline{AP} + \overline{OP} }}}} \approx 1 + 2\overline{\kappa } \frac{{\overline{AP} \times \overline{OP} }}{{\overline{AP} + \overline{OP} }}$$

Small curvatures stand here for radii of curvature larger than about ten times the typical distances $\overline{AP}$ and $\overline{OP}$ in the system. For instance, for our MBE412 growth chamber with $\overline{AP}$ and $\overline{OP}$ being in the half meter range, with *θ* = 0°, the relative error on γ_c_ when considering the linearization of Eq. (10) is 0.34% for a curvature of 0.1 m^−1^ (i.e. R = 10 m), and 1.37% for a curvature of 0.2 m^−1^ (R = 5 m) which, depending on the film and substrate materials and thicknesses, correspond to extremely stressed films, rarely observed in bottom-up fabrication processes. In standard conditions for semiconductor wafers, Eq. (5) also states that increasing $\left| {\overline{AP} } \right|$ and $\left| {\overline{OP} } \right|$ linearly increases the curvature measurement sensitivity.

With a rotating substrate in tilted configuration (Fig. 1b), it is possible to extract with a reasonable accuracy the absolute virtual flat reference pixel distance *d*_*0*_ of a curved surface using Eq. (11), in order to get a good insight on the absolute curvature. Consider $\overline{{\gamma_{Ct} }}$ and $\overline{{\gamma_{Cs} }}$, the magnification factors of a luminous object measured by the camera, respectively in the tangential and sagittal directions. If they are averaged over a complete revolution of the reflecting surface over ${\Psi }$ (in order to account for any deformation anisotropy of this surface), and averaged over two positions of the object, separated with a rotation angle of π/2 around the incidence axis (in order to account for any asymmetry of the object in sagittal and tangential planes), then, the curvatures deduced from the magnification factors in tangential and sagittal directions (Eqs. 6, 7) have to be equal. So, we obtain: $\frac{{\overline{{\kappa_{s} }} \left( \theta \right)}}{{\overline{{\kappa_{t} }} \left( \theta \right)}} = \frac{{\frac{{\overline{{\gamma_{Cs} }} - 1}}{{\overline{{\gamma_{Cs} }} }}}}{{\frac{{\overline{{\gamma_{Ct} }} - 1}}{{\overline{{\gamma_{Ct} }} }}}}\cos^{2} \theta = 1$ with $\overline{{\gamma_{Ct} }} = \frac{{d_{t} }}{{d_{0} }}$ and $\overline{{\gamma_{Cs} }} = \frac{{d_{s} }}{{d_{0} }}$, where *d*_*t*_ and *d*_*s*_ are the size in the image of the object in the tangential and sagittal planes respectively. This leads to:

11 $$d_{0} = \frac{{d_{s} d_{t} \sin^{2} \theta }}{{d_{s} - d_{t} \cos^{2} \theta }}$$

## Results

### Anisotropy measurements

**Figure 3:**
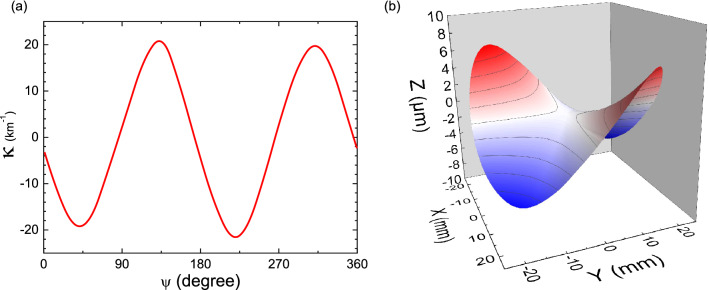
The curvature measured on a bare 50 mm diameter, 350 µm thick GaAs (001) oriented wafer while rotating at 12 rpm around its axis, at 580 °C (**a**) allows for its complete shape reconstruction in real-time (**b**).

Alignments are far less critical for the MIC technique than for laser deflectometry-based techniques. No operator’s adjustments have to be made between or during runs and it is possible to measure the curvature continuously during the whole substrate rotation, even when the substrate is wobbling, thus at any angle ${\Psi }$ around its axis (Fig. 1a). Figure 3a shows the curvature measured out of a 1.6 × 1.6 cm^2^, 5 × 5 luminous spots matrix facing the center of a bare standard 50 mm diameter, 350 µm thick (001) oriented GaAs wafer in normal incidence. This continuous measurement ability allows to access to the same measurement temporal statistics on a rotating wafer than on a non-rotating wafer as any curvature change at a given angle ${\Psi }$ can be compared to the initial curvature measured at the same angle before the film process. It is also possible to get a complete measurement of any anisotropic deformation as shown in Fig. 3b where the shape of this GaAs wafer is extrapolated from the curvature as a function of ${\Psi }$ given in Fig. 3a. In this example, the wafer presents an anisotropic shape that is consistent with a saddle-like deformation, within the supplier’s specifications (warp ≤ 10 µm)^33^. The measured deformation of the bare wafer can be set as the initial magnification at every ${\Psi }$ angle ($\gamma_{c} = 1$) before growth, and a fine determination of the deformation at any angle ${\Psi }$ can be performed.

### Precision

**Figure 4:**
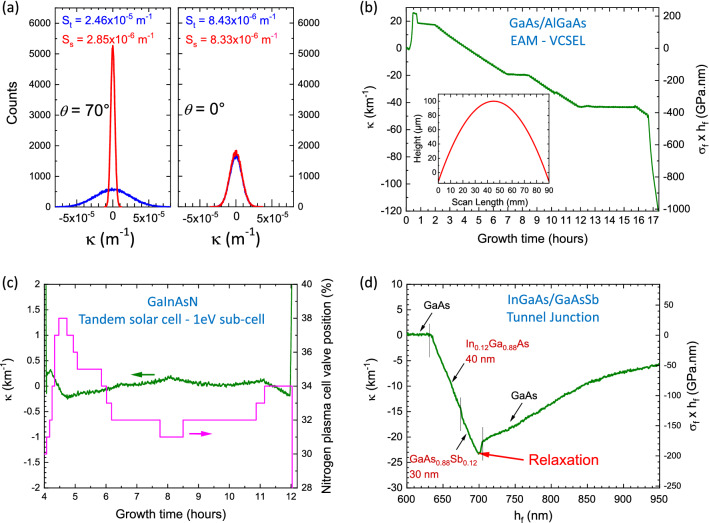
(**a**) Measurement of the curvature κ in tangential (blue) and sagittal (red) planes of a flat mirror repeated 10^5^ times, and mounted on an optical vibration-free bench in order to get insights on the precision of our MIC-based tool. The distances $\overline{OP}$ and $\overline{AP}$ are those of the configuration in our MBE412 system (0.516 m and 0.678 m respectively). The incidence angle is either *θ* = 70° (left) or *θ* = 0° (right). The object is composed of a 1.6 × 1.6 cm^2^, 5 × 5 matrix of luminous spots. (**b**–**d**) In situ measurements of the curvature change during MBE growths. (**b**) Complete growth of a ~ 10 µm thick, 17 h long GaAs/AlGaAs EAM-VCSEL. The insert is the height scan of the grown structure with respect of the bare wafer measured by a P15 + KLA Tencor. (**c**) Focus on the controlled growth of the 1 eV GaInAsN sub-cell alloy of a tandem solar cell. The variation of the opening of the nitrogen plasma cell valve is shown in pink (right scale). (**d**) Focus on a InGaAs/GaAsSb tunnel junction where a strain relaxation is observed when the total structure grown thickness is ~ 700 nm.

The precision of our MIC-based setup, has been tested on an optical bench using a flat 1″, 6 mm thick fused silica Al coated mirror acting as the reflecting surface. In the same configuration as for our MBE412 chamber ($\overline{OP}$ = 0.516 m, $\overline{AP}$ = 0.678 m), the curvature measurement standard deviations in transverse and sagittal planes measured are S_t_ = 8.43 × 10^–6^ m^−1^ and S_s_ = 8.33 × 10^–6^ m^−1^ for *θ* = 0°, and S_t_ = 2.46 × 10^–5^ m^−1^ and S_s_ = 2.85 × 10^–6^ m^−1^ for *θ* = 70° (Fig. 4a).

### In situ experiments

We have tested the MIC system in various conditions, as an in situ and real-time stress sensor during thin film grown by MBE, magnetron sputtering, CVD, as well as during plasma dry etching. Here, we present selected results obtained in our Riber MBE412 growth chamber, the MIC tool being mounted on its 70° “ellipsometer viewports” (Fig. 1b). We performed all the growths discussed here on standard 650 µm thick GaAs (001) oriented wafers, held by a molybdenum block thanks to their gravity as avoiding any clamping is mandatory for curvature measurements. Note that we observed it was also possible to perform measurements on wafers soldered with indium on a molybdenum block. Curvature changes can then be followed above the melting temperature of indium (156.6 °C). For each experiment, we measured a single wafer, rotating at 12 rpm around its axis.

The curvature is inferred from the pseudo-magnification factor γ_*c*_ by measuring the size of the virtual image of the object (Fig. 1c) at a rate of 100 Hz and dividing it by its size at the beginning of the experiment at any angle ${\Psi }$. The stress-thickness product is then calculated considering the biaxial modulus of GaAs (119.8 GPa), the thickness of the wafer, and $\overline{OP}$ and $\overline{AP}$ distances (0.516 m and 0.678 m respectively). Our MIC system was aligned just once at installation on the MBE412 viewports, and no further alignments were needed during months of experiments, making it a plug and play, easy to use, routine tool.

Figure 4b shows the curvature change during the complete growth of an Electro-Absorption Modulator (EAM)-VCSEL^17^, lasting 17 h for about 10 µm growth of about 3000 different (Al)GaAs layers (including digital alloys). From our knowledge, it’s the first time such continuous measurements of complex structures are reported due to three main constraints: long measurements, continuous reflectivity changes and relatively huge curvature change. The curvature induced by the growth (about 120 km^−1^) was crosschecked by comparing pre-growth and post-growth measurements of the wafer with a stylus profilometer (KLA Tencor P15 +). The spherical-like shape in a diameter of the wafer obtained is shown in the insert of Fig. 4b. We deduced many parameters from the curvature change measurement, as, for instance, doping levels, average alloy compositions, effusion cells flux drifts. Curvature rising and dropping at the beginning (first half hour) and the end (last half hour) of the run is due to temperature changes from 100 °C (load/unload temperatures) to 600 °C (growth temperature), giving access to the measurement of the difference of thermal expansion coefficient of AlAs and GaAs.

Measuring the curvature change in real-time ensures fine-tuning of growth parameters as illustrated by Fig. 4c. Ga_1-x_In_x_As_1-y_N_y_ quaternary alloy is a well-known candidate for the 1 eV sub-cell of multi-junction solar cells^19,20,34^. The perfect alloy should have ~ 6.5% In and ~ 2.3% N contents in order to be lattice-matched with the GaAs substrate^35^. Our MBE412 machine is equipped with a valved RF plasma cell where the valve opening allows for atomic nitrogen flux to be finely modulated at a given plasma cell condition (RF power and N_2_ gaz flow). We observed that the atomic nitrogen flux may drift during long periods of growth (typically 8 h for 3 µm thickness for the GaInAsN absorption layer). Mastering growth of such a lattice-matched alloy is a challenge, as even a small composition deviation would lead to strain and possibly to its plastic relaxation through dislocations in the complete structure, compromising the performance of the total solar cell. The MIC-based tool allows to successfully grow such layers with 100% success, as any shift of the nitrogen flux can be corrected in real-time by slightly changing the position of the nitrogen cell valve when any change in curvature of the whole structure^36,37^ is observed. The automation of this process is in progress.

MBE multilayer growth processes usually avoid plastic strain relaxation as they introduce defects as non-radiative centers for instance. More often, growers deal with poorly known plastic relaxation critical thicknesses^38,39^. Knowing which layer did actually relax (its position in the stack of layers) during the growth of complex multilayers is mandatory when growing materials close to these critical thicknesses’ limits. For instance, we observed the plastic strain relaxation during the growth of a 40 nm Ga_0.88_In_0.12_As/30 nm GaAs_0.88_Sb_0.12_ tunnel junction^40^. Figure 4d shows the curvature variation during this growth process, which is no longer monotonous at *h*_*f *_≈ 700 nm, while the GaAsSb layer was grown. We concluded that this tunnel junction was too thick for these alloys’ contents. Another hint that plastic relaxation occurred is the curvature change observed during the growth of the GaAs capping layer showing tensile stress (positive curvature change slope from *h*_*f*_ ≈ 705 nm), although the GaAs capping layer should show no curvature change if no relaxation had occurred.

The growth of new candidate alloys can sometimes be tricky, as for example for bismide alloys^41^. Using the MIC-based tool coupled with RHEED analysis unravels atomistic original phenomena driving the level of bismuth incorporation in the growing layer and thus driving the growth as reported in ref.^41^.

## Discussion

We demonstrate here the capabilities of the MIC method as an alternate way to measure the curvature of reflective surfaces. It is based on a simple optical phenomenon which can be observed by anyone in everyday life, when using a concave magnifying mirror for instance, but have surprisingly not been exploited for scientific measurements. The original formalism developed here could have been the key element limiting the development of such measurements. Instead, hard-to-use and limited laser deflectometry-based tools have been developed, which however are based on a well-established optical formalism. The following discussion highlights some key differences between the two techniques and how the MIC technique overcome most of the limitations of laser deflectometry-based techniques without backing on precision and accuracy.

Note that the reader which compares our method to others, projecting images on the surface for which they follow their deformation would be misled as for those techniques, only non-reflecting surfaces can be imaged and the optical formalism is completely different: here, the reflecting surface is part of the optical system creating the analyzed image.

Based on reported works, the main differences between the MIC and laser deflectometry-based techniques should be the capability of the MIC-based one to measure curvature at any incidence angle. Indeed, increasing incidence angle induces an anisotropy in the deformation of the image between sagittal and tangential planes, associated to astigmatism. The optical formalism for MIC is developed, even outside Gauss’ conditions, to take into account this astigmatism (see Eqs. (6) and (7)), thanks to early twentieth century textbooks^29,30^. This opens the possibility to use a MIC-based tool in specular conditions, even at high incidence angles.

**Figure 5:**
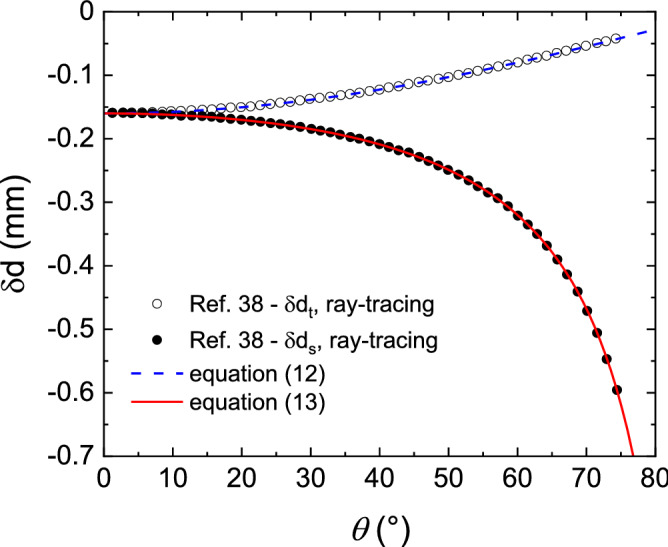
Laser deflectometry spots spacing change (*δd*) versus incidence angle *θ* on a convex surface (R = − 10 m): comparison between ray-tracing simulations made in reference ^42^ (open and plain circles in tangential and sagittal planes respectively) and Eqs. (12) and (13) (blue dash and red continuous curves respectively) with the parameters set in reference ^42^ (L = 0.8 m, d = 1 m).

Astigmatism as also been reported for laser deflectometry-based techniques^42^.The usual equation, developed to deduce the curvature out of a laser deflectometry measurement, only accounts for the astigmatism in the sagittal plane (equation (13) in ref.^42^). Based on the developments of Eqs. (6) and (7), we propose the following set of equations in the tangential and sagittal planes, for laser deflectometry:

12 $$\overline{{\kappa_{t} }} \left( \theta \right) \approx \frac{{\delta d_{t} }}{{d_{t} }}\frac{1}{2L\cos \theta }$$

13 $$\overline{{\kappa_{s} }} \left( \theta \right) \approx \frac{{\delta d_{s} }}{{d_{s} }}\frac{\cos \theta }{{2L}}$$

where *θ* is the incidence angle, measured with respect of the reflecting surface normal, *L* is the optical path length from the reflecting surface to the CCD detector, *d*_*t*_ and *d*_*s*_ the distances between adjacent incident laser beams and *δd*_*t*_ and *δd*_*s*_ the changes in the spacing between the adjacent spots on the CCD detector in the tangential and sagittal planes respectively. Although Eq. (13) has been validated in ref.^42^, one can see in Fig. 5 that the set of equations developed here seems to perfectly fit the data points given in reference^42^, giving a good hint on the validity of our formalism to account for astigmatism when the incidence angle is not null.

This means that laser deflectometry could also be used at any incidence angle if using Eqs. (12) and (13) to account for astigmatism. Of course, a geometric mean of these equations leads to the expression of the curvature that would be independent of the incidence angle:

14 $$\overline{\left| \kappa \right|} = \sqrt {\overline{{\kappa_{t} }} \left( \theta \right)\overline{{\kappa_{s} }} \left( \theta \right)} \approx \frac{1}{2L}\sqrt {\frac{{\delta d_{t} \delta d_{s} }}{{d_{t} d_{s} }}}$$

For *θ* = 0°, Eq. (12) or (13) can be written in terms of a measured magnification defined as $\gamma_{L} = \left( {d + \delta d} \right)/d$:

15 $$\gamma_{L} \approx 1 + 2\overline{\kappa }L$$

**Figure 6:**
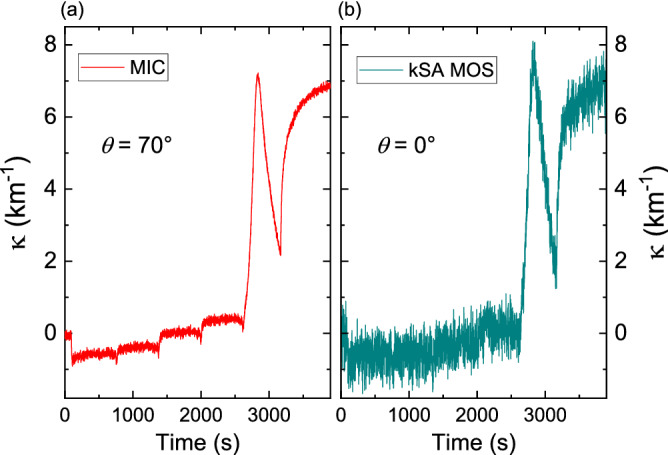
Curvature changes during a sequence of growths and growth stops of sputter-deposited Ag on a-Si as a function of time measured simultaneously with our MIC-based setup (**a**) and a commercial laser deflectometry-based tool (**b**). The MIC-based setup was mounted on the 70° viewports of the magnetron sputtering chamber while the kSA MOS was mounted at normal incidence (*θ* = 0°).

Comparing Eqs. (10) and (15) suggests laser deflectometry is theoretically about twice as sensitive to curvature as MIC (when considering equivalent distances for distances $\overline{AP}$, $\overline{OP}$ and *L*) for small curvatures. Nevertheless, we measured a better ultimate precision with our MIC tool (Fig. 4a) than the ones given in laser deflectometry-based equipment’s documentations (5 × 10^–4^ m^−1^ for Laytec EpiCurve TT and 2 × 10^–5^ m^−1^ for kSA MOS^43,44^ at normal incidence). We attribute this result mainly to our state-of-the-art image analysis algorithm that takes advantage of the latest computer capabilities. The question that arises is: would our image analysis algorithm turn the most precise laser deflectometry-based tool to an even more precise one? In our opinion, the ultimate precision obtained in a protected environment (vibration-free optical bench) might be improved, but the ultimate precision is not the only limiting factor for an actual real-time in situ measurement, as many external parameters may degrade the curvature measurements precision. The ability of the different techniques to measure precisely in a vibrating environment also depends on their robustness, which is a major strength of the MIC technique. In order to illustrate this, we made coupled experiments using a state-of-the-art laser deflectometry-based tool (kSA MOS) and our MIC setup. We could then directly compare their precision in a given vibrating environments, during the same experiments. The MOS tool was mounted on the normal incidence viewport of a magnetron sputtering chamber while the MIC setup was mounted on its *θ* = 70° “ellipsometer viewports” (configuration of Fig. 1b). The MOS was carefully aligned to the sample before each experiment in order for the CCD sensor to capture the full spots matrix, and to avoid the laser beams to be deviated or diffracted by any sample surface irregularities. No such alignment prior to any experiment was made with the MIC tool except the initial one, at system installation, which only consisted on focusing the camera to the virtual image of the spots matrix, and to roughly tilt the camera to align the spots matrix to the center of the acquired images. For comparison purposes, both MOS and MIC tools used a 3 × 3 spots matrix, the dimensions of the matrix of the MIC plate being 3 × 3 mm^2^. Moreover, the MIC camera acquisition frequency and thus its data acquisition rate, was lowered to 30 Hz. Curvature changes were measured during growth and growth stops sequences of Ag on an oxidized 100 µm thick, 10 × 10 mm^2^ Si wafer. Figure 6a shows the curvature variations measured with the MIC setup in the sagittal plane during this experiment, whereas Fig. 6b shows the curvature changes measured with the MOS simultaneously. The same data filtering was applied to display the two curves (1 Hz). The four plateaus before t = 2500 s correspond to four growth and growth stop sequences, that can clearly be observed with the MIC setup, while they are drowned in the noise for the data set obtained with the MOS. In this environment, and in the particular experimental conditions described above, the curvature standard deviation is measured in this experiment to be 3.71 × 10^–4^ m^−1^ for the MOS, and 7.4 × 10^–5^ m^−1^ for the MIC, making it about 5 times more sensitive. Even though the titled configuration of the MIC setup increases its sensitivity by a factor $1/\cos \theta$ which, for *θ* = 70°, is about 2.92, the MIC is a more precise tool whatever the incidence angle. Nevertheless, the standard deviations measured in this vibrating environment are more than an order of magnitude larger than the ultimate ones obtained for our MIC setup (Fig. 4a), or for the MOS^44^, suggesting that the MIC robustness that we pointed out for the alignment of the tool is also a key element when it comes to in situ and real-time measurements inside a process chamber.

Moreover, the MIC tool is able to measure and analyze the data at a rate of 100 Hz. Even though measuring at high rate does not improve the standard deviation of the MIC instrument, it can improve the final measurement precision by a factor $\sqrt N$ when temporal averaging is performed over *N* points. To our knowledge, laser deflectometry-based systems usually work at lower rates (5–30 Hz).

Note that there is no limit in the shape of the luminous object for the MIC measurements. We choose to use luminous spots ordered in a matrix for convenience as its image analysis is easy and robust to stray lights for instance. The more spots, the more measurement points, the more precise is the final measurement.

Even using the theoretical upgrade of the laser deflectometry-based techniques given above (Eqs. 12, 13), allowing accurate measurements at any incidence angles, there are many advantages to use MIC rather than laser deflectometry:

- The MIC tool is more precise than commercially available laser deflectometry-based tools.
- MIC can be used with any light source. This has many advantages:
  - In luminous environments like plasma deposition or etching chambers, it is possible to choose a range of wavelength for the luminous object such that light perturbations from other sources are minimal. Wavelength filters can also be used for this purpose.
  - When using a white light source, MIC is fairly insensitive to reflectivity changes from the studied surface (see Fig. 4b). This is a major advantage if one wants to follow thin film processes, where thickness variations induce reflectivity changes.
- Many advantages come from the fact that the MIC technique is based on the use of a camera with its lens, and so it is focused on a virtual image that is beyond the surface of interest.
  - This makes MIC to be robust to slight tilts or wobbling of the studied surface. So, we just align the system once at installation, and no further alignment is needed during an experiment, or between experiments. To our experience, this is not the case for laser deflectometry, where constant adjustments have to be made even during experiments in order to keep the laser beams on the CCD sensor. This robustness also allows for the continuous measurement on rotating wafers, and thus a complete determination of any anisotropy that could build up.
  - For the MIC imaging technique, the camera lens is focused close to the virtual image, thus at about twice the distance AP. Any ghost image coming from reflections of light on viewports would be out of focus, so easy to get rid of. With lasers beams, users must take great care not to detect unwanted reflections on viewports, and expensive tilted viewports must sometimes be used to overcome this issue.
  - The MIC imaging technique is less sensitive to flakes that may fall on viewports, which is very likely to happen in growth systems like MBE chambers. A laser beam can be blocked or scattered by a single flake, whereas the virtual image detected by a camera would be fainted. Its detection would then be more robust to flakes.
  - The size of the MIC object is not constrained to a CCD sensor as for laser deflectometry. It is then possible to make it larger, the only limit being the size of the wafer itself, or the size of the viewport for in situ measurements, but could be as big as needed for ex-situ curvature measurements of very large samples.
  - A final advantage to use imaging rather than laser beams deflectometry is that imaging is less sensitive to surface irregularities. An impurity or defect on the reflecting surface might scatter a laser beam, and care has to be taken before each measurement in order to check that no laser spot is vanished due its interaction with a surface defect. Tedious alignments must then be performed before any measurement sequence with a laser deflectometry-based tool. Because of the MIC imaging method, the virtual image is far less sensitive to surface defects, as the camera is focused close to the virtual image.

Another point which deserves attention is that the magnification variation with curvature (Eq. 10) diverges for concave curvature $\overline{\kappa } = \left( {\overline{AP} + \overline{OP} } \right)/\left( {2\overline{AP} \times \overline{OP} } \right)$. The closer to this diverging point, the more sensitive the system is. One could use this property to get an extremely sensitive system around the diverging point.

One may anticipate that the main drawback of the MIC technique could arise from its non-linearity at high curvatures, where laser deflectometry is still linear. However, Eqs. (6) and (7) analytically account for the non-linearity to extract the exact curvature. Non-linearity only becomes an issue when considering a too curved surface as a flat reference before a deposition or etching process. In this particular case, thanks to Eq. (11), the measurement of the absolute curvature should be performed before the process in order to avoid quantitative errors. Moreover, it is not an issue for semiconductor wafers as their curvatures are rarely large enough for the non-linearity to be significant. For instance, an extreme curvature of − 0.2 m^−1^ which would induce a non-linearity of about 1.4% in the configuration of our MBE412 system, would correspond to the epitaxial growth of about 100 µm of AlAs, or of about 7.5 µm of Ga_0.8_In_0.2_As on a 650 µm thick GaAs wafer, which are thicknesses far beyond the critical values for plastic relaxation for these materials^45,46^. This curvature also corresponds to a 25 µm thick standard SiO_2_ stressed film (− 100 MPa^47^) on a 650 µm thick Si wafer. Such a huge SiO_2_ thickness is rarely met in the semiconductor industry.

## Conclusion

A new precise and accurate, robust, and easy-to-use curvature measurement system has been depicted, based on basic optical principles, namely magnification by a mirror. An original optical formalism has been developed to address curvature measurements at any incidence angle, proofed using Zemax OpticStudio ray tracing simulations. The potentialities of the method both for in situ and real-time measurements in tough industrial conditions, including measurements of the anisotropy of a semiconductor wafer and its absolute curvature have been demonstrated. The MIC technique addresses the major limitations encountered by laser deflectometry-based systems such as strong reflectivity dependence of monochromatic sources, precise and continuous adjustments needs, limited precision and process drifts for very long processes.

The MIC-based tool opens the possibility to push further the industrial automation of complex growth processes (VCSEL, solar cells…), the understanding of phenomena driving the growth of complex or very thin structures (e.g. quaternary or bismide alloys, EAM-VCSELs, 2D films…) or the measurement of physical constants very accurately (e.g. thermal expansion coefficients).

## Data Availability

The datasets generated during and/or analyzed during the current study are available from the corresponding author on reasonable request.
